# First report on knockdown resistance mutations in wild populations of *Aedes aegypti* from Argentina determined by a novel multiplex high-resolution melting polymerase chain reaction method

**DOI:** 10.1186/s13071-023-05840-y

**Published:** 2023-07-06

**Authors:** Alberto N. Barrera-Illanes, María Victoria Micieli, Marina Ibáñez-Shimabukuro, María Soledad Santini, Ademir J. Martins, Sheila Ons

**Affiliations:** 1 Laboratorio de Neurobiología de Insectos (LNI), Centro Regional de Estudios Genómicos, Facultad de Ciencias Exactas, Universidad Nacional de La Plata, CENEXA, CONICET, La Plata, Buenos Aires Argentina; 2 Laboratorio de Insectos Vectores, Centro de Estudios Parasitológicos y Vectores (CEPAVE CONICET CCT-La Plata-UNLP), La Plata, Buenos Aires Argentina; 3 Instituto Nacional de Parasitología “Dr. Mario Fatala Chaben”, ANLIS-Malbran, Ministerio de Salud de La Nación, CONICET, Ciudad Autónoma de Buenos Aires, Buenos Aires, Argentina; 4 Laboratório de Fisiologia e Controle de Artrópodes Vetores, Instituto Oswaldo Cruz (Fiocruz), Rio de Janeiro, Brazil

**Keywords:** Dengue, Vector management, Mosquito, Insecticide, Pyrethroid resistance, Arbovirus

## Abstract

**Background:**

The mosquito *Aedes aegypti* is an urban vector of dengue and other arboviruses. During epidemics of these viruses, pyrethroid insecticides are used for the control of adult mosquitoes. The worldwide resistance of *Ae. aegypti* to these insecticides is a cause of failure of vector control campaigns. The primary target of pyrethroids is the voltage-gated sodium channel. Point mutations in the gene coding for this channel, called knockdown resistance (*kdr*) mutations, are associated with pyrethroid resistance. Two *kdr* mutations, V1016I and F1534C, have increased in frequency in natural populations of *Ae. aegypti* in the Americas during the last decade. Their association with pyrethroid resistance has been largely demonstrated in field populations throughout the Americas, and in in vitro assays. Diagnostics for *kdr* polymorphism allow early detection of the spread of insecticide resistance, which is critical for timely decisions on vector management. Given the importance of resistance management, high-throughput methods for *kdr* genotyping are valuable tools as they can be used for resistance monitoring programs. These methods should be cost-effective, to allow regional-scale surveys. Despite the extensive presence of *Ae. aegypti* and incidence of dengue in Argentina, the presence, abundance, and distribution of *kdr* mutations in populations of this mosquito have yet to be reported for the country.

**Methods:**

*Aedes aegypti* samples were collected as immature stages or adults from Buenos Aires Metropolitan Area and northern localities of Tartagal (Salta Province) and Calilegua (Jujuy Province). Immature stages were maintained in the laboratory until they developed into adults. A high-resolution melting assay, based on an analysis of melting temperatures, was developed for the simultaneous genotyping of V1016I and F1534C *kdr* mutations. We used this method to infer the presence and frequencies of *kdr* alleles in 11 wild populations from Argentina.

**Results:**

We demonstrated the presence of *kdr* mutations in *A*e*. aegypti* in Argentina in regions where this species is under different selection pressures due to the use of pyrethroids. The populations under analysis are located in geographically distant regions of the species’ distribution in Argentina: the northern provinces of Salta and Jujuy and the Buenos Aires Metropolitan Area. Higher frequencies of resistant-associated alleles were detected in the northern region. We report a multiplex high-throughput assay based on a high-resolution melting polymerase chain reaction method for the simultaneous genotyping of V1016I and F1534C *kdr* mutations. This assay was shown to be cost-effective, and thus provides an interesting molecular tool for *kdr* genotyping in *A. aegypti* control campaigns.

**Conclusions:**

We report, to the best of our knowledge for the first time, the presence of *kdr* mutations in populations of *Ae. aegypti* from geographically distant locations of Argentina that differ with respect to their epidemiological situation and history of mosquito control. We have developed a high-throughput method for the genotyping of *kdr* mutations in *Ae. aegypti* from the Americas. Given its affordability and short running time, this method can be used in control campaigns to monitor the presence and spread of *kdr* alleles. The information provided here is relevant for the rational design of control strategies in the context of integrated vector management.

**Graphical Abstract:**

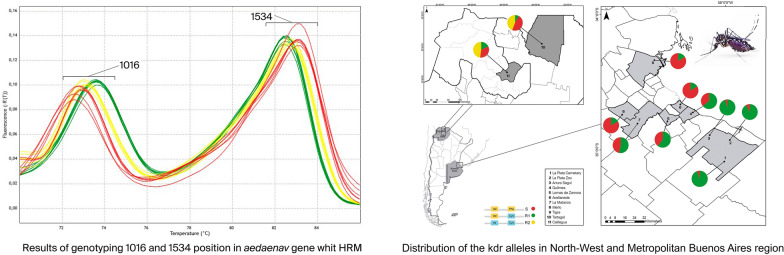

**Supplementary Information:**

The online version contains supplementary material available at 10.1186/s13071-023-05840-y.

## Background

Around 390 million people worldwide are infected with dengue every year. In the past decades, the number of reported cases in the Americas has increased more than tenfold, to more than 16 million, from the 1.5 million cases recorded in the 1980s [1]. Dengue, Zika, chikungunya, and the re-emerging virus yellow fever, are transmitted by females of the mosquito species *Aedes aegypti*, which is considered one of the most successful invasive species worldwide [2]. Urbanization, human population growth and climate change are factors that promote the expansion and abundance of *Ae. aegypti* [3]. This mosquito was reintroduced into Argentina in 1986 and it is now established in all the northern and central provinces of the country; its southern boundary is formed by the provinces of Buenos Aires [4], Neuquén [5] and Río Negro [6]. Since *Ae. aegypti*’s reintroduction, four major and widely distributed dengue epidemics have occurred in Argentina (in 2009, 2016, 2020 and 2023), which were of considerable public health concern. There were more than 93,000 cases in the most recent outbreak, which occurred in 2023 (https://www.argentina.gob.ar/salud/epidemiologia/boletines).

Control of *Ae. aegypti* expansion involves environmental management through the elimination of domestic and peri-domestic breeding sites and the use of larvicides such as insect growth regulators, organophosphate neurotoxins and the biolarvicide *Bacillus thuringiensis israelensis* [7]. It is recommended that the use of adulticides should be restricted to periods of arbovirus epidemics [7]; for these, the neurotoxic pyrethroids are the preferred compounds, given their favorable toxicological properties. Even though the organophosphate malathion is used in some countries in cases of high pyrethroid resistance, its application for vector control is banned in Argentina, and pyrethroids are the only insecticides allowed in the country for domestic use and public health applications [8].

The target site of pyrethroid insecticides is the voltage-gated sodium channel (Na_v_), a membrane protein present in excitable cells. Given their rapid lethal action, the effect of pyrethroids is known as knockdown; the single nucleotide polymorphisms of the sodium channel gene that confer resistance to pyrethroids are known as knockdown resistance (*kdr*) mutations [9]. In the Americas, four *kdr* mutations related to the loss of pyrethroid susceptibility have been identified in *Ae. aegypti*: Val to Leu in position 410 (V410L), Ile to Met in position 1011 (I1011M), Val to Ile in position 1016 (V1016I), and Phe to Cys in position 1534 (F1534C) [10–12]. The frequency of I1011M seems to have decreased in the last 20 years, and its role in the pyrethroid resistance of natural populations is not clear [13]. Conversely, in the last decade, V1016I and F1534C mutations have increased in frequency in natural populations in the Americas [14–16]. In addition, 410L^*kdr*^ is strongly associated with 1016I^*kdr*^ and 1534C^*kdr*^ in field populations. Results of a recent study [12] showed that 410L^*kdr*^ augmented resistance to a pyrethroid in the field, with a significant interaction with spraying application distance.

With respect to positions 1016 and 1534, three alleles of the Na_v_ gene of *Ae. aegypti* (aedaenav) are widely distributed in South and North America [14, 16, 17]: 1016V + 1534F (susceptible); 1016 V + 1534C^*kdr*^ (resistant 1; R1); 1016^*kdr*^ I + 1534C^*kdr*^ (double mutation, resistant 2; R2). The R3 allele (1016I^*kdr*^ + 1534F) was detected with a very low frequency (≤ 0.1% of the samples) in Brazil [10], Mexico [15] and Florida [17]. Conversely, the R1 and R2 genotypes are predominant in all the regions studied in the Americas.

The role of *kdr* mutations in pyrethroid resistance in *Ae. aegypti* has been well established. In the last 10 years, *kdr* alleles in natural populations propagated in parallel with increasing levels of resistance to pyrethroids [16–18]. More recently, a comparison of the homozygous R1R1, R2R2 and the heterozygous R1R2 laboratory lines of *Ae. aegypti* with a SS susceptible line confirmed that the three *kdr* genotypes conferred deltamethrin resistance to the insects, and that the R2R2 genotype conferred the highest level of resistance [19]. Electrophysiological recordings from *Xenopus laevis* oocytes showed reduced affinity to type I (permethrin) but not type II (deltamethrin) pyrethroids in oocytes expressing the R1 allele (1016V + 1534C^*kdr*^) sodium channel compared to those expressing the SS allele (1016V + 1534F) [20]. In parallel, channels expressing the R3 (1016I^*kdr*^ + 1534F) allele did not present reduced sensitivity to pyrethroids when expressed in *X. laevis* oocytes. Moreover, when the R2 allele was expressed in *X. laevis* oocytes, a higher resistance to both type I and type II pyrethroids was observed compared to when the R1 allele was expressed [21].

Resistance monitoring is critical for the success of vector control campaigns, and should be undertaken for operational decision making. Diagnostics for *kdr* polymorphisms allow early detection of the spread of insecticide resistance, given that they can be used to detect an individual carrying the mutation before it is fixed in the population through the selection pressure exerted by the insecticide. Studies on *kdr* distribution and abundance have been performed in the Americas, e.g. Venezuela [22], Mexico [14], USA [16] and Brazil [18, 19], for resistance monitoring. Despite the extensive presence of *Ae. aegypti* and the incidence of dengue in Argentina, the existence, abundance and distribution of *kdr* mutations in this mosquito have yet to be reported for the country.

Given the importance of resistance management, cost-effective and high-throughput methods for *kdr* genotyping are considered invaluable for resistance monitoring programs. For *Ae. aegypti*, the methods that have been implemented to date are based either on allele-specific polymerase chain reaction (PCR) [23] or on the use of TaqMan probes [24]. While the running costs associated with allele-specific PCR are minor compared to those associated with TaqMan assays [25], the results of the former may be less reproducible than those of the latter when the assays are carried out by different laboratories, given the need for careful setting up of the PCR conditions to avoid amplification of nonspecific products. On the other hand, high-resolution melting (HRM) is a reproducible probe-free high-throughput method for genotyping. Well-calibrated equipment and a third generation fluorescent double-stranded DNA dye are used in this method [26]. HRM has economic and practical advantages with respect to other methods for the detection of *kdr* in *Anopheles gambiae* [25]. Single-site HRM was used for genotyping *kdr* mutations in Asian populations of *Ae. aegypti* [27], but the development of a multiplex HRM-based assay (mHRM) for *kdr* genotyping would allow the genotyping of two mutations in a single reaction tube, which would decrease both costs and running time.

Here, we report, to the best of our knowledge for the first time, the presence of *kdr* mutations in *Ae. aegypti* from Argentinean populations under different selection pressure with pyrethroids. To detect these, we developed a mHRM assay for the simultaneous genotyping of sites 1016 and 1534, which may be a valuable tool for the monitoring of *kdr* in *Ae. aegypti* mosquitoes from the Americas.

## Methods

### Mosquito sampling and DNA extraction

**Fig. 1:**
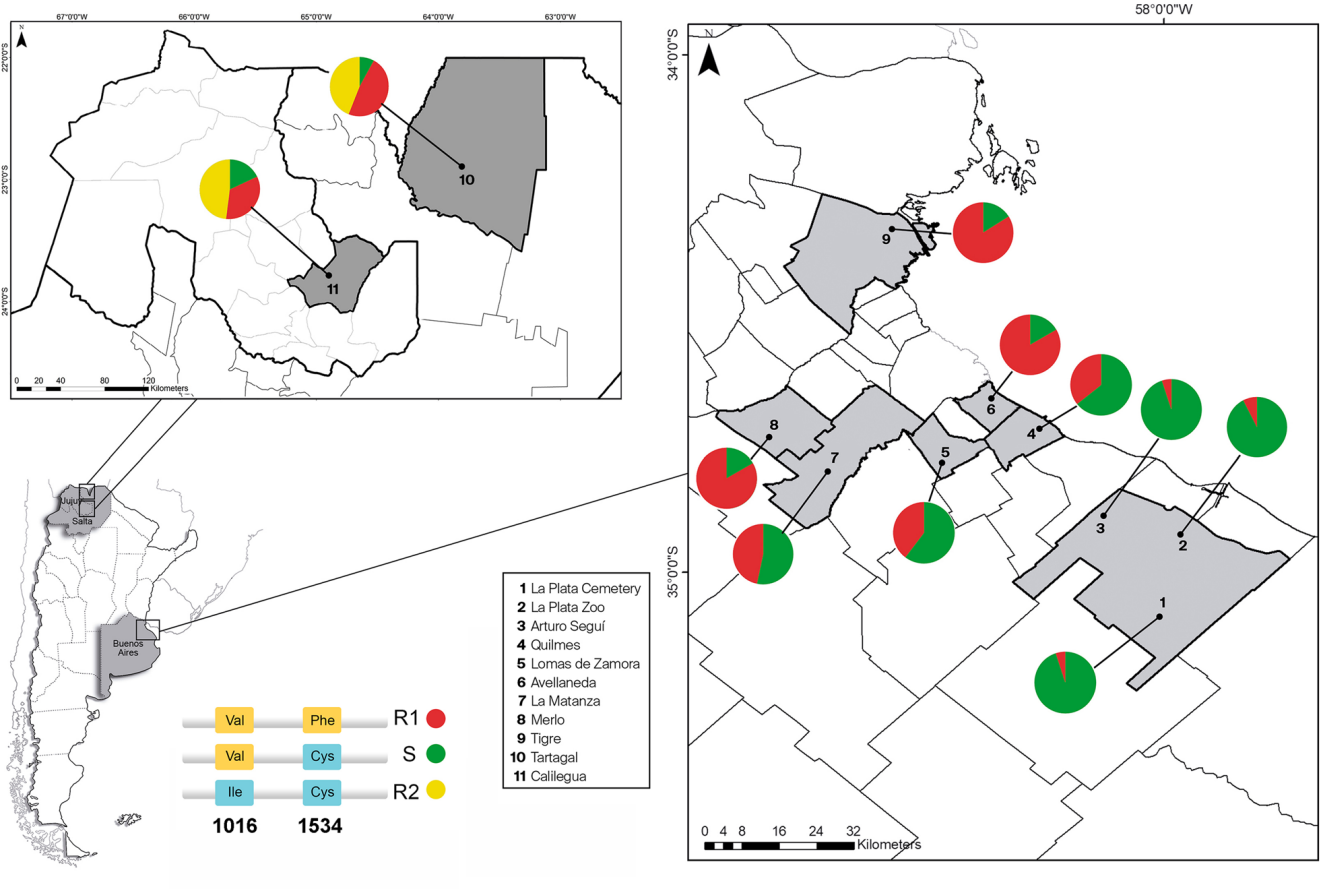
Distribution of the knockdown resistance (*kdr*) alleles in *Aedes aegypti* in Tartagal and Calilegua National Park. Tartagal (Salta Province) and Calilegua National Park (Jujuy Province) (inset, upper left); Buenos Aires Metropolitan Region (inset, right)

Mosquito sampling was performed in 2018 and 2019 in the provinces of Salta (Tartagal; S22°30′58.9ʺ, W63°48.079′), Jujuy (Calilegua National Park; S23°38′20″, W64°50′17) and Buenos Aires [La Plata (S34°55′17.2ʺ, W 57°57.272ʹ); Merlo (34°39′55″S, 58°43′39″O); Arturo Seguí (34°53′16″S, 58°07′36″O); Lomas de Zamora (34°46′00″S, 58°24′00″O); Avellaneda (34°40′00″S 58°21′00″O); Quilmes (34°43′00″S, 58°16′00″O); La Matanza (34°43′00″S, 58°38′00″O) and Tigre (34°25′00″S, 58°35′00″O)] (Fig. 1). These localities were selected to compare geographically distant regions within the distribution of *A. aegypti* to estimate the dispersion of *kdr* mutations in the country. We also considered the history of dengue epidemics and the consequent control of adult mosquitoes through spatial spraying with adulticides, which has been carried out more recently in Buenos Aires Metropolitan Area (Área Metropolitana de Buenos Aires; AMBA) than in the northern provinces. Furthermore, AMBA is the most populated region of the country, and thus has a greater number of inhabitants that are potentially exposed to arboviruses.

The tested mosquitoes were captured as immature stages or adults from the field. Immature stages were collected from artificial containers. All immature stages were reared to adults in the laboratory facilities of Centro de Estudios Parasitológicos y de Vectores (CEPAVE). Adults were captured in the field with aspirators, nets, or traps. Morphological identification was determined under a stereoscopic microscope by using dichotomous taxonomic keys [28]. Mosquito samples were conserved in ethanol at − 80 °C until genomic DNA was extracted from individual mosquitoes. For DNA extractions, an adapted protocol based on magnetic nanoparticles was used [29]. DNA from each individual was eluted in 50 µL of pure water.

### Development of the HRM assay

Primer v.0.4.0 software (http://bioinfo.ut.ee/primer3–0.4.0/primer3/) was used to design the primers flanking the IIS6 and IIIS6 Na_v_ segments, which include positions 1016 and 1534, respectively, in the aedaenav gene (AAEL023266; see primer sequences in Table 1). Amplicon melting temperatures (MTs) were predicted with uMELT online software (https://www.dna.utah.edu/umelt/umelt.html). GC tails were added to forward and reverse primers of the 1534 amplicon to differentiate at least 2 °C MT of both amplicons. Both the real-time PCR and HRM steps were performed on an ArialMx Real-Time PCR system (Agilent). PCR was performed in 12 μL containing 6 μL Brillant HRM Ultra-fast Loci Master Mix (Agilent), 200 nM of each primer and 2 μL of genomic DNA diluted 1/10. The PCR amplification protocol began with a denaturation step at 95 °C for 3 min, followed by 40 cycles of 5 s denaturation at 95 °C and 30 s annealing-extension at 62 °C. After amplification, the reaction tubes were cooled to 55 °C, then warmed from 55 to 95 °C at the rate of 0.2 °C/s. The melting curves were analyzed with AriaMX 1.5 software (Agilent). For the setting up of the reaction, samples previously genotyped by Dr. A. J. Martin’s lab from Brazilian populations were used: SS (1016 VV + 1534 FF), SR2 (1016 VI^*kdr*^ + 1534 FC^*kdr*^) and R2R2 (1016 I^*kdr*^ I^*kdr*^ + 1534 C^*kdr*^ C ^*kdr*^). Samples of known genotypes determined by Sanger sequencing were also included in every mHRM plate for comparisons with samples of unknown genotype.

**Table 1:** Sequence and application of the designed primers

Primer name	Sequence	Use
Ae1534qPCRFwGC2	CCGGCGGCGGTGTACCTCTACTTTGTGTTCTTCA	HRM and Sanger sequencing
Ae1534qPCRRvGC	CCGGCGGCGGCAGCGTGAAGAACGACCCG	HRM and Sanger sequencing
Ae1016G2_Fw^a^	CAAATTGTTTCCCACTCGCACAG	HRM and Sanger sequencing
Ae1016G3_Rv	CGTTTCGTTGTCGGCAGTCGGTG	Sanger sequencing
Ae1016qPCRRev^a^	AGCAAGGCTAAGAAAAGGTTAAG	HRM

*HRM* High-resolution melting

^a^Primer designed from the intronic region

### Sanger sequencing

Genotypes of random selected samples were confirmed by sequencing of the relevant regions of the aedaenav gene. For Sanger sequencing, the genomic DNA was used as a template for the PCR reactions using GoTaq (Promega). The cycling consisted of initial denaturation for 5 min at 95 °C, followed by 40 cycles of 30 s at 95 °C (denaturation) and 30 s at 62 °C, and a final extension step at 72 °C for 5 min. Twenty-one samples were sequenced for the 1016 amplicon and 14 for the 1534 amplicon by using the Sanger method at Macrogen (Seoul, Korea) with the Ae1016GqPCRRv and Ae1534qPCRFwGC primers, respectively (Table 1).

### Statistical analysis

One-way ANOVA followed by Tukey’s multiple comparison test was used to compare MTs among different variants for positions 1016 or 1534. The frequency of each mutation was calculated by adding the number of homozygotes multiplied by 2 to the number of heterozygotes and dividing the total by the sample size multiplied by 2. The 95% confidence interval was calculated using the adjusted Wald formula [30]. Hardy–Weinberg equilibrium was evaluated, and a chi-square test with 1 or 3* df* (when three or six genotypes were evidenced, respectively) was used to test the null hypothesis of equilibrium.

## Results

**Fig. 2:**
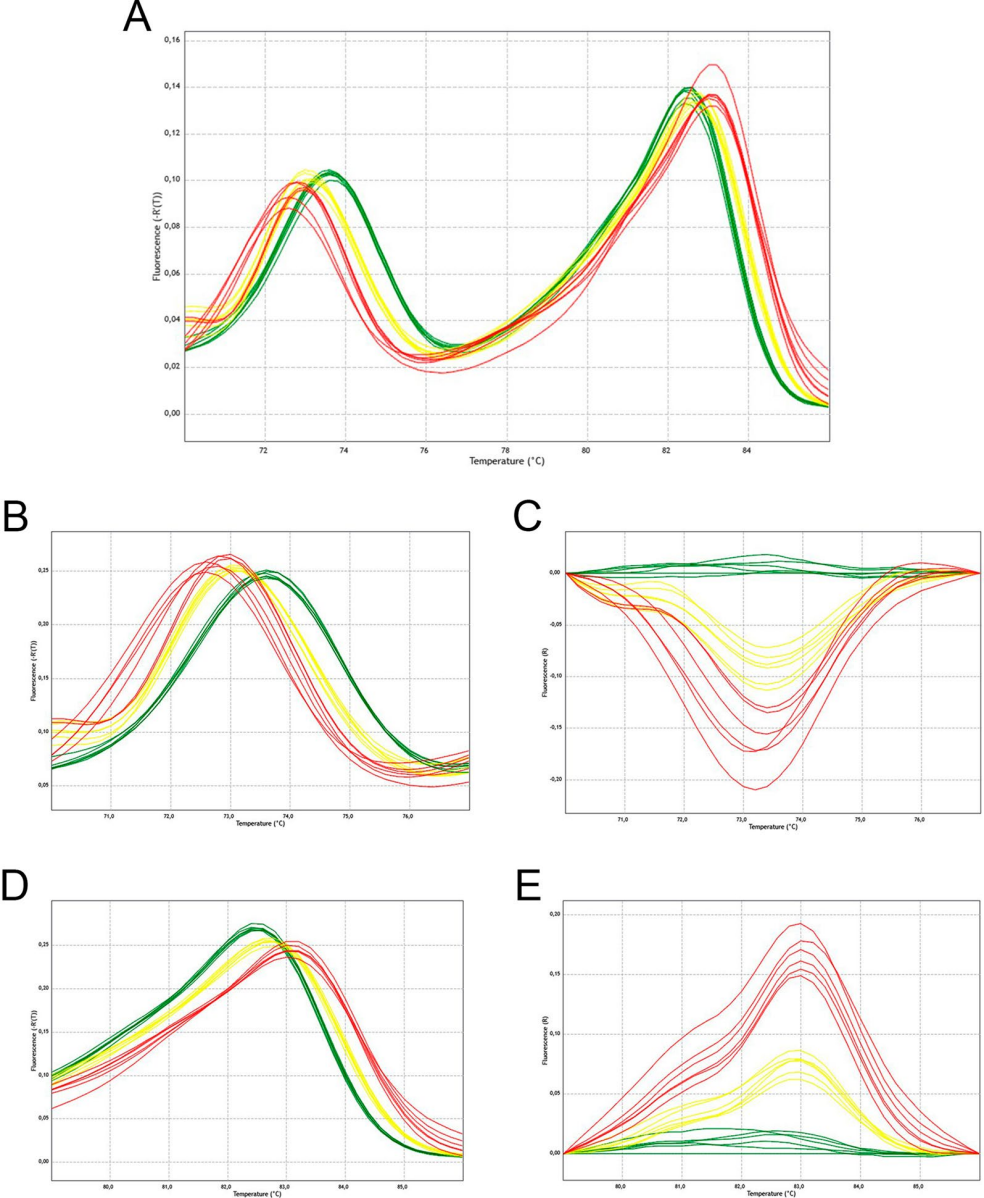
**a**–**e** Results of genotyping positions 1016 and 1534 in the voltage-gated sodium channel (Na_v_) gene of *Aedes aegypti* (*aedaenav*) with high-resolution melting (HRM). Detection of *kdr* single nucleotide polymorphism by melt curve analysis. Alleles are distinguished by changes in the melting temperature (MT). **a** Raw results of multiplex HRM (mHRM). Peaks on the left and on the right indicate, respectively, IIS6 and IIIS6 Na_v_ segment amplicons. **b**, **c** Derivative and difference melting plots for IIS6 with variation in the 1016 position. **d**, **e** Derivative and difference melting plots for IIIS6 with variation in the 1534 position.* Red* *kdr* homozygous standard (R2R2),* yellow* heterozygous standard (SR2),* green* wild type homozygous standard (SS)

Fragments containing the 1016 and 1534 positions of the aedaenav gene (vectorbase number AAEL023266) were simultaneously amplified in duplex PCR from genomic DNA, using two primer pairs in the reaction tube. By the addition of GC tails in the 5’ regions of forward and reverse primers flanking the 1534 position (Table 1), a difference of around 10 °C in the MT was achieved between 1016 (MT around 73 °C) and 1534 (MT around 83 °C) amplicons (Fig. 2a). This difference allowed the individual analysis of both polymorphic sites using two primer pairs in a single reaction. Figure 2a shows representative curves of homozygous samples in both positions (SS and R2R2), and the heterozygous SR2. For representative curves of the heterozygous SR1, R1R2 and the homozygous R1R1 see Additional file 1.

As expected for a substitution of a guanine (in the wild type 1016S) by an adenine (in the 1016I^*kdr*^ variant), the MT of the IIS6 amplicons was highest for the 1016VV genotype (73.61 °C ± 0.02 °C; *n* = 10) and lowest for 1016 I^*kdr*^I ^*kdr*^ (72.74 °C ± 0.07 °C; *n* = 6); for the heterozygote 1016 VI^*kdr*^, the MT was intermediate (73.09 °C ± 0.02 °C; *n* = 6) (Fig. 2b, c). On the other hand, for the IIIS6 segment, the MTs were 82.45 °C (± 0.01 °C, *n* = 6) for 1534 FF and 83.07 °C (± 0.01 °C, *n* = 12) for 1534 C^*kdr*^C^*kdr*^. The MT was intermediate for the heterozygote 1534 FC^*kdr*^ (82.68 °C ± 0.02 °C, *n* = 6) (Fig. 2d, e).

**Fig. 3:**
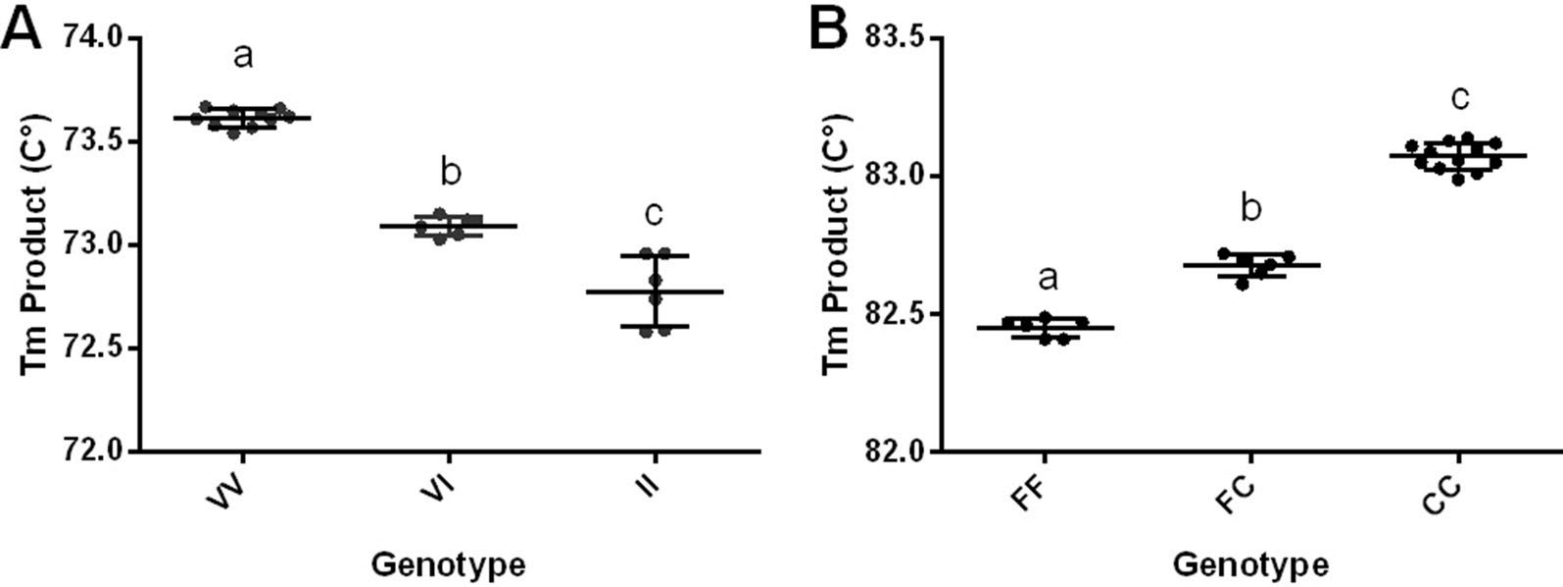
**a**, **b** Changes in MTs with variation in the position of Na_v_ in *Aedes aegypti*. **a** Position 1016. Homozygous Val (*VV*), present in genotypes SS, SR1 and R1R1; heterozygous Val Ile (*VI*), present in genotypes SR2 and R1R2; homozygous Ile (*II*), present in genotype R2R2.** b** Position 1534. Homozygous Phe (*FF*), present in genotypes SS; heterozygous Phe Cys (*FC*), present in genotypes SR1 and SR2; homozygous Cys (*CC*), present in genotypes R1R1, R1R2 and R2R2. Different letters indicate significant differences (*P* < 0.05; ANOVA, Tukey’s multiple comparison test)

MTs were obtained for the IIS6 and IIIS6 paired amplicons with their respective variations in the 1016 and 1534 positions, and significant differences were observed in the MTs for both positions between both the homozygous and the heterozygous samples (*P* < 0.05; *n* = 6–12) (Fig. 3). The genotyping of heterozygous samples was less straightforward for particular samples, and we observed that both the quality and quantity of DNA extracted from individual mosquitoes were key to accurate genotyping.

The mHRM method developed here presented advantages compared to the other options [24, 31], such as higher throughput, and a one-step and closed-tube reaction, given that both alleles can be detected in a single reaction (Table 2).

**Table 2:** Comparison of methods for the genotyping of* kdr* mutations in positions 1016 and 1534 (genotyping of 85 individuals for both positions)

	Multiplex allele-specific PCR^a^	TaqMan^b^	Singleplex HRM	Multiplex HRM
Equipment required	Thermocycler; electrophoresis system; transilluminator with camera; computer	Real-time thermocycler; computer	Real-time thermocycler; computer	Real-time thermocycler; computer; software
Equipment costs^c^ (USD)	9500	20,000	20,000	21,000
Time for completion	4.5 h	2.5 h	2.5 h	1.2 h
Characteristics	PCR followed by gel electrophoresis; multiplex	Closed tube; one independent reaction for each mutation	Closed tube; one independent reaction for each mutation	Closed tube; multiplex
Number of steps	2	2	2	1
Number of primers	7	4	4	4
Number of TaqMan probes	0	4	0	0
Number of tubes/wells required	96	192	192	96
Running costs (USD)	40	212	129	64

*PCR* Polymerase chain reaction, USD US dollars

^a^Saingamsook et al. [31]

^b^Macoris et al. [24]

^c^Costs (US dollars;* USD*) are estimated from prices in Argentina

We used mHRM for the analysis of *Ae. aegypti* from eight populations from the center of Argentina (AMBA) and two populations from the north-west (Tartagal-Salta Province and Calilegua National Park-Jujuy Province) (Fig. 1). R1 and S alleles were present at different frequencies in all the localities under study, whereas R2 was only found in the northern region (Tartagal and Calilegua) (Table 3; Fig. 1). To confirm the mHRM results by a gold standard sequencing method, a number of samples of genomic DNA were PCR amplified for IIS6 and IIIS6 Na_v_ segments with specific primers and subjected to direct Sanger sequencing. For the 1016 position, the results for all of the samples subjected to Sanger sequencing were in accordance with those obtained using mHRM (*n* = 21), whereas for position 1534, there was agreement between the Sanger sequencing and HRM results for 12 of 14 samples (the two samples that did not agree were for heterozygous samples). All the sequenced IIS6 fragments were identical, with the exception of samples from Tartagal that had the *kdr* substitution in the 1016 position (Additional file 2: A). In two samples from La Plata Zoo and one from Arturo Segui, a silent substitution of thymidine to cytosine in position 1528 (Additional file 2: B) was detected. This polymorphism in the 1528 position has been previously reported in samples from Africa and the Americas [15].

**Table 3:** Frequencies of genotypes for the V1016I and F1534C *kdr* mutations in *Aedes aegypti* from Argentina

Region	Province-locality	Type of population	Genotype frequencies (confidence interval)						*n*	HWE test	
			SS	SR1	R1R1	SR2	R1R2	R2R2		χ^2^	*P*-value
Center	La Plata Zoo-Buenos Aires	Peri-urban	**0.85** (0.69–1.00)	**0.15** (0.00–0.31)	**0.00** (0.00–0.11)	**0.00**	**0.00**	**0.00**	**20**	**0.13**	**0.72**
Center	La Plata Cemetery-Buenos Aires	Peri-urban	**0.90** (0.78–1.00)	**0.10** (0.00–0.22)	**0.00** (0.00–0.08)	**0.00**	**0.00**	**0.00**	**30**	**0.08**	**0.77**
Center	Arturo Seguí-Buenos Aires	Rural	**0.97** (0.87–1.00)	**0.03** (0.00–0.13)	**0.00** (0.00–0.08)	**0.00**	**0.00**	**0.00**	**30**	**0.01**	**0.93**
Center	Quilmes-Buenos Aires	Urban	**0.21** (0.06–0.36)	**0.71** (0.55–0.87)	**0.07** (0.00–0.19)	**0.00**	**0.00**	**0.00**	**28**	**5.88**	**0.02**
Center	Lomas de Zamora-Buenos Aires	Urban	**0.31** (0.15–0.47)	**0.59** (0.42–0.76)	**0.10** (0.00–0.22)	**0.00**	**0.00**	**0.00**	**29**	**1.47**	**0.23**
Center	Avellaneda-Buenos Aires	Urban	**0.00** (0,00–0.08)	**0.33** (0.17–0.49)	**0.67** (0.50–0.83)	**0.00**	**0.00**	**0.00**	**30**	**1.20**	**0.27**
Center	La Matanza-Buenos Aires	Urban	**0.17** (0.03–0.30)	**0.73** (0.58–0.88)	**0.10** (0.00–0.22)	**0.00**	**0.00**	**0.00**	**30**	**6.72**	**0.01**
Center	Merlo-Buenos Aires	Urban	**0.00** (0.00–0.08)	**0.33** (0.17–0.49)	**0.67** (0.51–0.83)	**0.00**	**0.00**	**0.00**	**30**	**1.2**	**0.27**
Center	Tigre-Buenos Aires	Urban	**0.00** (0.00–0.08)	**0.32** (0.16–0.48)	**0.68** (0.51–0.84)	**0.00**	**0.00**	**0.00**	**28**	**1.09**	**0.30**
North West	Calilegua-Jujuy	Silvatic	**0.04** (0.00–0.15)	**0.00** (0.00–0.09)	**0.12** (0.00–0.26)	**0.28** (0.11–0.45)	**0.44** (0.29–0.62)	**0.12** (0.00–0.26)	**25**	**7.08**	**0.07**
North West	Tartagal-Salta	Urban	**0.00** (0.00–0.09)	**0.00** (0.00–0.09)	**0.24** (0.08–0.40)	**0.28** (0.01–0.31)	**0.48** (0.30–0.66)	**0.12** (0.00–0.26)	**25**	**9.05**	**0.03**

*HWE* Hardy–Weinberg equilibrium,* SS* 1016VV + 1534FF;* SR1* 1016VV + 1534FC,* R1R1* 1016VV + 1534CC,* SR2* 1016VI + 1534FC,* R1R2* 1016VI + 1534CC,* R2R2* 1016II + 1534CC

For AMBA, we analyzed samples from both rural/peri-urban locations and populated urban localities. In the former, the most common allele was S (98.3% in Arturo Seguí; 95% in La Plata Cemetery and 92.5% in La Plata Zoo) (Fig. 1; Table 3). In the urban localities, a higher frequency of R1 was detected, although it was the least common allele in Quilmes (31.7%), but the most common one in Tigre (73.4%). In the other localities analyzed, the rate of the R1 allele was around 50% (55.4% in Lomas de Zamora, 55.6% in Avellaneda, 48.1% in La Matanza and 53.85% in Merlo) (Fig. 1; Table 3). In Tartagal, a locality with a more intense use of pyrethroids historically, the R2 allele was detected in 36.0% of the samples, but the most common allele was R1 (56.0%); the S allele was found in only 8.0% of the individuals. A similar pattern was detected in Parque Nacional Calilegua; the R2 allele was present in 48% of the samples, the R1 allele in 34%, and the S allele in only 18%. Genotype frequencies, confidence intervals for the six possible genotypes in each population and the results of the tested deviation from Hardy–Weinberg equilibrium are presented in Table 3. For both the La Matanza and Quilmes populations, from AMBA, the assumption of Hardy–Weinberg equilibrium was rejected (*P* < 0.05), as the heterozygous SR1 genotype for both populations was higher than expected according to the hypothesis. For the northern populations, the Hardy-Weinberg equilibrium was rejected for Tartagal (*P* < 0.05), as R1R2 was the most abundant genotype. R1R2 was also the most abundant genotype in Calilegua, but the Hardy–Weinberg equilibrium hypothesis was not rejected in this case (*P* = 0.07).

## Discussion

Pyrethroid resistance in *Ae. aegypti* seriously compromises dengue control campaigns. Alternative adulticides, such as the organophosphate malathion, are not permitted for the treatment of human dwellings in Argentina, given environmental and sanitary considerations [8]. The rational design of vector control campaigns needs to include resistance-management strategies to prolong the period of efficacy of pyrethroids. In this context, the monitoring of resistance-conferring alleles, such as *kdr* mutations, should be routinely performed to aid decision-making for control campaigns. Notwithstanding the wide distribution of *kdr* alleles in natural populations of *Ae. aegypti* worldwide, and the epidemiologic situation in Argentina, the presence and/or distribution of *kdr* have not been previously reported for this country.

There are a number of assays available for genotyping *kdr* alleles in *Ae. aegypti*. The most widely used one of these for populations in the Americas is based on TaqMan probes [24]. Allele-specific PCR has also been developed for this; although cheaper, it has a lower throughput, and the reaction conditions, which must be carefully determined to avoid inaccurate results, were not reproducible in different labs [25]. Singleplex HRM has been used to genotype mutations in Asian populations of *Ae. aegypti* [31], which achieved double the genotyping effort when compared with the mHRM proposed here. Multiplex allele-specific PCR may be a suitable option for less well-equipped laboratories, though the costs of skilled labor should be taken into consideration. All life stages of mosquitoes (from eggs to adults), and even dead individuals, can be sampled during routine surveillance, without necessitating the collection or rearing of insects. Using a high-throughput genotyping technique such as the mHRM presented here, results can be obtained in a few hours (Table 2). Careful sample conservation and the use of high-yielding DNA extraction methods positively affect the quality of the results. Furthermore, we observed that it is important to use a high-quality HRM master mix, given that preliminary assays with other commercially available options gave a number of uninterpretable results. Also, it is important to calibrate the qPCR equipment with the same reagents that will be used for the assays. Where genotyping results are ambiguous, which was mainly found for heterozygous samples, a complementary method (such as TaqMan probes or Sanger sequencing) can also be used. In sum, the use of available complementary methods, including the mHRM presented here, will improve genotyping efforts in terms of time and cost.

Despite the lack of detailed information on insecticide application by region in Argentina, we can assume that there is a direct relationship between dengue epidemics and the control of adult mosquitoes by spatial spraying with adulticides, as recommended by the National Health Ministry during dengue outbreaks. The use of pyrethroids was prolonged in the northern region (Salta and Jujuy) because dengue epidemics have been recorded there since 1998 [32], with cases reported almost every year [33] up until the present (Bulletin of the Ministry of Health Argentina 2023). Accordingly, we observed a higher frequency of the R2 genotype both in a sylvatic and in an urban collection site in this region. In agreement with these findings, Harburger et al. [34] reported pyrethroid resistance in *Ae. aegypti* adults from Argentina for the first time in Salta Province (Salvador Mazza). Given that the Calilegua mosquitoes were collected in a protected sylvatic area, where insecticides are not used, it is possible that individuals invaded the national park from nearby urban locations.

AMBA has a more recent history of dengue outbreaks, which represented 35% of the most important dengue outbreaks registered in Argentine in 2016 [35], 2020 and 2023 (Bulletin of the Ministry of Health Argentina), despite it being the most populated region of Argentina. Interestingly, a positive correlation exists between the specific years of treatment with pyrethroids and the emergence of the 1016I^*kdr*^ mutation. Also, rural or peri-urban populations that were not treated with pyrethroids during ultralow volume spraying had a higher proportion of the wild type (sensitive) allele. These results are in agreement with the sequential selection of *kdr* mutations in *Ae. aegypti* [21]. Given that the V1016I^*kdr*^ mutation alone does not confer insecticide resistance, it is possible that mutation F1534C^*kdr*^ emerges earlier in response to selective pressure due to pyrethroid use. The 1016I^*kdr*^ + 1534C^*kdr*^ alleles, which emerged more recently in the Americas, lead to a greater and broader spectrum of pyrethroid resistance.

Although 1016I*kdr* and 1534C*kdr* are mutations of high epidemiological relevance, other* kdr* mutations that have an impact on pyrethroid sensitivity have been described for *A. aegypti* [36]. Among these, 410L^*kdr*^, detected in samples from Brazil, Mexico and USA, was strongly associated with the 1016I^*kdr*^ and 1534C^*kdr*^ alleles [10–12], indicating that it may have a wide distribution throughout the Americas. Given the strong association of the 410L^*kdr*^ allele with 1016I^*kdr*^ and 1534C^*kdr*^, it may not be necessary to genotype the 410 site for resistance monitoring purposes [10]. However, the frequency of 410L^*kdr*^ over time and throughout the Americas, and its association with other* kdr* mutations and with levels of insecticide resistance, are all of relevance for the study of pyrethroid resistance in mosquitoes [12]. The setting up of mHRM conditions for the simultaneous detection of three or more single nucleotide polymorphisms is challenging, yet feasible [37]. Hence, further studies should be undertaken for the development of mHRM that can simultaneously detect 410L^*kdr*^, 1016I^*kdr*^, 1534C^*kdr*^ and/or other polymorphisms of interest in the *Ae. aegypti* sodium channel gene.

Even though the frequency results obtained here should be interpreted with caution due to the low number of individuals analyzed, they indicate, to our knowledge for the first time, the extensive presence of *kdr* alleles in field populations of *Ae. aegypti* from Argentina. To confirm this finding, further, nationwide, studies are necessary. However, we have shown here that these alleles are present in regions that are more than 1500 km apart, which suggests that they have a wide distribution.

Information on *kdr* alleles in *Ae. aegypti* populations in Buenos Aires and northern provinces of Argentina should be taken into consideration in the rational design of vector control campaigns. The results presented here, and especially those for the populations sampled from the densely populated AMBA region, should be taken as a warning sign when designing *Ae. aegypti* control campaigns. Despite the fact that the rate of insecticide resistance for a given population will be influenced by its entire genetic background, including metabolic resistance mechanisms, detected *kdr* mutations are useful molecular markers for the early detection of pyrethroid resistance in vector control campaigns. In this context, we have developed a new high-throughput method that may have economic and practical benefits for the management of insecticide resistance in *Ae. aegypti* in many regions of the Americas. In the context of the lack of approved alternative compounds, decision-making with regard to planned pyrethroid treatments should be both careful and rational to enable their utility to be prolonged.

## Conclusions

We report here, to our knowledge for the first time, the presence of *kdr* mutations in *Ae. aegypti* populations from geographically distant regions of Argentina, which have different epidemiological situations and different histories of mosquito control efforts. We have developed a high-throughput multiplex HRM method for the genotyping of these mutations. The method developed here could be incorporated into programs designed to assess the presence of resistance-associated alleles and control their spread, and may lead to reduced costs and running time. Optimal results would be obtained through the use of complementary, available genotyping methods. It is important to use these methods to obtain a complete picture of the frequencies of *kdr* alleles throughout the area of distribution of *Ae. aegypti* in Argentina for the effective short-term management of resistance, and to remain vigilant in assessing the dynamics of these frequencies over the longer term. In parallel, scientific effort needs to be urgently focused on the development of novel vector control alternatives which should have a low environmental impact.

### Supplementary Information

**Additional file 1: ** Detection of *kdr* single nucleotide polymorphism by melt curve analysis. Raw curves showing both 1016 (left peak) and 1534 (right peak) positions. *Red kdr* homozygous standard (R2R2), *yellow* heterozygous standard (SR2), *green* homozygous standard (SS). **A** Representative curve for the SR1 genotype (*violet*). **B** Representative curve for the R1R1 genotype (*blue*). **C** Representative curve for the R1R2 genotype (*grey*).

**Additional file 2. **Clustal alignments of sequences obtained by Sanger sequencing and gene AAEL023266 in Vectorbase for regions 1016 and 1534.

## Data Availability

All data generated or analyzed during this study are included in this published article (and its supplementary information files).
