# Correction: First report on knockdown resistance mutations in wild populations of Aedes aegypti from Argentina determined by a novel multiplex high-resolution melting polymerase chain reaction method

**DOI:** 10.1186/s13071-023-05894-y

**Published:** 2023-08-14

**Authors:** Alberto N. Barrera-Illanes, María Victoria Micieli, Marina Ibáñez-Shimabukuro, María Soledad Santini, Ademir J. Martins, Sheila Ons

**Affiliations:** 1 Laboratorio de Neurobiología de Insectos (LNI), Centro Regional de Estudios Genómicos, Facultad de Ciencias Exactas, Universidad Nacional de La Plata, CENEXA, CONICET, La Plata, Buenos Aires, Argentina; 2 Laboratorio de Insectos Vectores, Centro de Estudios Parasitológicos y Vectores (CEPAVE CONICET CCT-La Plata-UNLP), La Plata, Buenos Aires, Argentina; 3 Instituto Nacional de Parasitología “Dr. Mario Fatala Chaben”, ANLIS-Malbran, Ministerio de Salud de La Nación, CONICET, Buenos Aires, Argentina; 4 Laboratório de Fisiologia e Controle de Artrópodes Vetores, Instituto Oswaldo Cruz (Fiocruz), Rio de Janeiro, Brazil

**Correction: Parasites & Vectors (2023) 16:222** **https://doi.org/10.1186/s13071-023-05840-y**

Following publication of the original article [1], the authors flagged that there was an error in Fig. 1: in the bottom-left of the figure, the green and red circles were the wrong way around. The figure has now been corrected in the published article (so the red circle is now by 'R1', while the green circle is now by 'S'). The authors thank you for reading this erratum and apologize for any inconvenience caused.
