# Supplementary material for: First report on knockdown resistance mutations in wild populations of Aedes aegypti from Argentina determined by a novel multiplex high-resolution melting polymerase chain reaction method
Source: Parasit Vectors. 2023 Jul 6;16:222. doi: 10.1186/s13071-023-05840-y (PMC10324241; doi:10.1186/s13071-023-05840-y)

additional file 1: Detection of *kdr* single nucleotide polymorphism by melt curve analysis. Raw curves showing both 1016 (left peak) and 1534 (right peak) positions Red: *kdr* homozygous standard (R2R2); Yellow: heterozygous standard (SR2); Green: wild-type homozygous standard (SS). **A:** Representative curve for the SR1 genotype (violet). **B:** Representative curve for the R1R1 genotype (blue). **C:** Representative curve for the R1R2 genotype (grey).

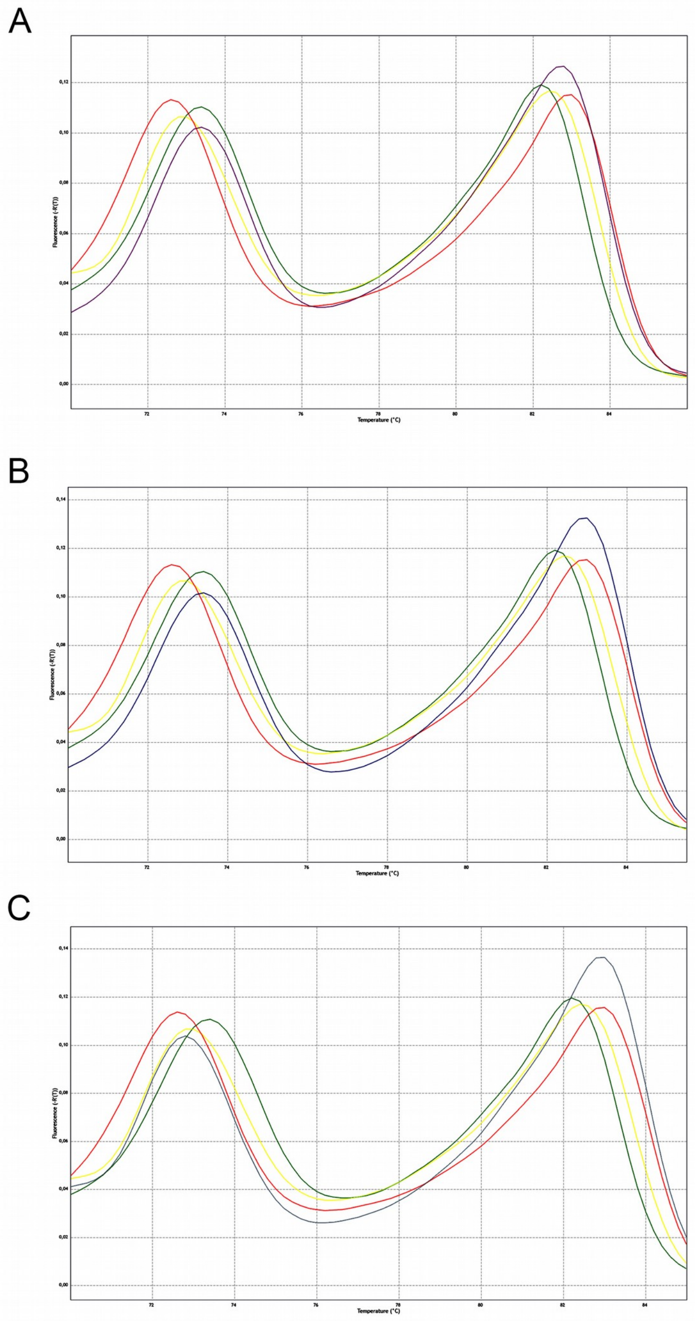

Supplement: Supplementary file 1 — Additional file 1: Detection of kdr single nucleotide polymorphism by melt curve analysis. Raw curves showing both 1016 (left peak) and 1534 (right peak) positions. Red kdr homozygous standard (R2R2), yellow heterozygous standard (SR2), green homozygous standard (SS). A Representative curve for the SR1 genotype (violet). B Representative curve for the R1R1 genotype (blue). C Representative curve for the R1R2 genotype (grey). [file 13071_2023_5840_MOESM1_ESM.pdf]
